# Role of microRNA in chronic lymphocytic leukemia onset and progression

**DOI:** 10.1186/s13045-015-0112-x

**Published:** 2015-02-20

**Authors:** Veronica Balatti, Yuri Pekarky, Carlo M Croce

**Affiliations:** Department of Molecular Virology, Immunology and Medical Genetics, Comprehensive Cancer Center, Wexner Medical Center, The Ohio State University, Columbus, OH USA

**Keywords:** microRNA, CLL

## Abstract

B-cell chronic lymphocytic leukemia (CLL) is the most common human leukemia occurring as indolent or aggressive form. CLL clinical features and genetic abnormalities are well documented, but molecular details are still under investigation. MicroRNAs are small non-coding RNAs involved in several cellular processes and expressed in a tissue-specific manner. MicroRNAs regulate gene expression, and their deregulation can alter expression levels of genes involved in development/progression of tumors. In CLL, microRNAs can function as oncogenes or tumor suppressors and can also serve as markers for CLL onset/progression. Here, we discuss the most recent findings about the role of microRNAs in CLL and how this knowledge can be used to identify new biomarkers and treatment approaches.

## CLL: characteristics and outcomes

Chronic lymphocytic leukemia (CLL) is the most common human leukemia. This disease can arise in two forms, aggressive and indolent, both characterized by the accumulation of incompetent CD5+ B lymphocytes [1]. Unfavorable prognosis is associated with the expression of unmutated immunoglobulin heavy variable genes (UM-IgH-V_H_) and high level of 70 kD zeta-associated protein (ZAP-70). Chromosomal alterations are detected in >80% of cases and can discriminate patients with different outcomes [2,3]: (i) low risk, normal karyotype or 13q deletion; (ii) intermediate risk, 11q deletion or trisomy 12; and (iii) high risk, 17p deletion or complex karyotype [4]. The ability of malignant cells to respond to microenvironmental stimuli via B-cell receptor (BCR) signaling, interaction with accessory cells or microvescicles identified in plasma has recently emerged as another factor in the clinical course of CLL [5,6].

### Signatures of microRNAs in CLL cells and microenvironment

MicroRNA profiles can distinguish normal B cells from malignant CLL cells and have been associated with prognosis, progression, and drug resistance [7]. A signature profile describing 13 microRNAs able to differentiate aggressive and indolent CLL was reported [8]. Patients with high *miR-21* and *miR-155* had a higher risk of death compared to patients with low expression of these microRNAs [9,10], and *miR-181b* can predict time to treatment, acting as a biomarker of progression [11]. MicroRNA signatures can also predict refractoriness to fludarabine [7]. *MiR-148a*, *miR-222*, and *miR-21* exhibited a higher expression in non-responders while lower levels of *miR-34a* were observed in resistant subjects even in absence of p53 aberrations [12]. Recent studies demonstrated that microRNAs are involved in an intricate interplay with BCR signaling and microenvironmental stimuli. Indeed, BCR signaling and immunoglobulin production can be regulated by microRNAs [13] while the expression of certain microRNAs can be altered via BCR stimulation [5]. A signature of 39 differentially expressed miRNAs was found upon BCR stimulation [14]. BCR activation can lead to reduced levels of *miR-29c*, *miR-150*, *miR-181b*, or *miR-223* [15], and low expression of these microRNAs was observed in patients with shorter survival and/or time to treatment [16]. *MiR-155* is instead upregulated in response to BCR ligation and appears to play a role during T- and B-cell development [5,13]. Recent reports proposed that miRNAs are released by donor cells through circulating microvescicles (MVs) [6]. Extracellular miRNAs are present in the plasma of CLL patients at different levels from healthy controls. Since therapy often reduced the pool of malignant cells but does not affect plasma features, it is likely that MVs continue to induce abnormal gene profiles. Therefore, miRNA profiling in plasma could reflect another mechanism of malignant B-cell proliferation [17].

### Role of microRNAs in CLL

Recently, researchers have focused on the molecular impact of deregulation of microRNA expression in CLL. *MiR-15/16* cluster, *miR-34b/c*, *miR-29*, *miR-181b*, *miR-17/92*, *miR-150*, and *miR-155* family members, the most deregulated microRNAs in CLL, were found to regulate important genes, helping to clarify molecular steps of disease onset/progression.

#### MicroRNA-15a/16-1

In 2002, a cluster of two microRNA genes, *miR-15a* and *miR-16-1*, was located within the 13q14.3 deleted region [18]. 13q14.3 deletion in CLL, the most frequent genomic aberration, associates with the longest treatment-free interval [19]. Accordingly, *miR-15a/16-1* expression was found downregulated in ~66% of CLL cases [18]. The importance of *miR-15a/16-1* was confirmed in a study in New Zealand black (NZB) mice, the only mouse strain that naturally develops CLL [20]. A point mutation causing a decrease of *miR-16-1* expression in NZB lymphoid tissues and elevated levels of Bcl-2 was found in the *miR-15a/16-1* precursor (located in the mouse genomic region homologous to 13q14) [20]. B-cell lymphoma 2 (*BCL2*) is a central player in the genetic program of eukaryotic cells, promoting survival by inhibiting apoptosis [21], and it is overexpressed in many human cancers [22]. *MiR-15a/16-1* and *BCL2* expression levels were found inversely correlated in CLL [23], and downregulation of these microRNAs in leukemic cell lines resulted in an increase of Bcl2 expression with consequent inhibition of apoptosis [23]. Moreover, microarray experiments performed on CLL patients with high vs low levels of *miR-15a/16-1* identified a gene signature which also contains *MCL1*, an antiapoptotic *BCL-2* family member associated with B-CLL cell survival and chemotherapy resistance [24].

The first genetic manipulation in mice that confirmed the importance of *miR-15a/16-1* deletion in CLL was carried out by Dr. Dalla-Favera and colleagues [25]. These authors designed a model with conditional alleles that either resembled the loss of the minimal deleted region (Mdr), spanning entirely the host gene *Dleu2* gene [26], or the specific *miR-15a/16-1* cluster deletion, without altering the expression of *Dleu2* [25]. Mdr knockout (KO) animals lived less than wild-type (WT) siblings and succumbed to leukemia, while the differential survival between *miR-15a/16-1* KO and their WT littermates was not statistically significant, indicating that the latter were affected by a milder phenotype than the former [25].

Additional factors regulate *miR-15a/16-1* expression besides chromosomal deletion. Veronese et al. identified an allele-specific transcription mechanism. Normally, one allele of *miR-15a/16-*1 is transcribed by RNA polymerase II together with *DLEU2* and the other by RNA polymerase III independently of the host gene. In 13q14 deleted patients, exclusive RNA polymerase III-driven transcription was observed and found to associate with high expression of *ZAP70*. Indeed, in a CLL case of monozygotic twins that differed in *ZAP70* status and clinical features, transcription of *primiR-15a/16-1* was driven by RPIII in the aggressive *ZAP70*-positive patient and by RPII in the indolent *ZAP70*-negative case [27]. Furthermore, CLL cells show a reduced amount of processed intermediates *pre-miR-15a/16-1*, while the precursor *pri-miR-15a/16-a* was not decreased, indicating a block of miRNA maturation at the DROSHA processing step. Interestingly, the mRNA levels of pri-miRNA processing cofactors were not decreased, and CLL cells retained the ability to cleave other microRNA precursors, suggesting that DROSHA processing is specifically impaired for *mir-15a/16-1* and possibly other specific miRNAs [28]. Lastly, the two copies of the critical region were found to replicate asynchronously. Differential replication timing represents an early epigenetic mark resulting in different chromatin packaging and monoallelic expression. These findings support a model of consecutive inactivation of both alleles by a series of different mechanisms, resulting in a final complete inactivation. Indeed, in the majority of CLLs, monoallelic deletion is sufficient for complete loss of *mir-15a/16-1* function rather than a twofold downregulation. Inactivation of the active chromosome copy by epigenetic mechanisms could therefore be a tumor-initiating event [29].

#### *MicroRNA-34b/c* and *microRNA-34a*

**Figure 1:**
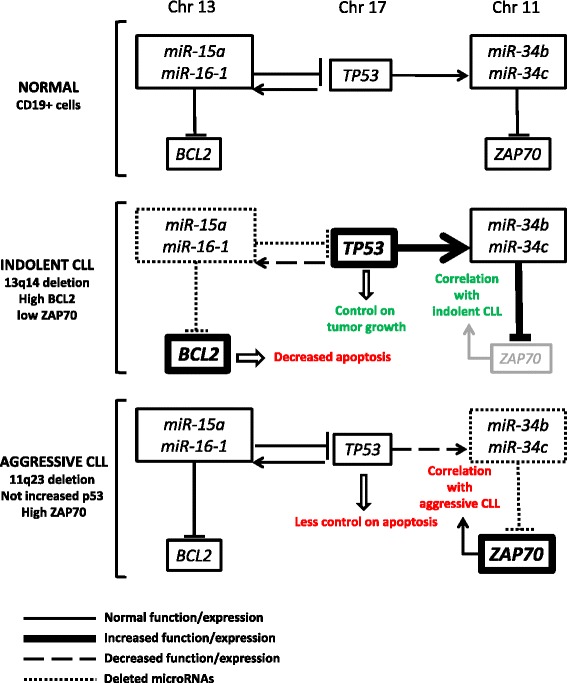
**Interplay between microRNA expression and chromosomal aberration in CLL.**

11q deleted region includes the *miR-34b/c* cluster locus [30], while deletion of 17p leads to abrogation of the p53 tumor suppressor [31], and 13q deletion involves *miR15a/16-1* downregulation. To verify possible interactions between these chromosomal alterations, we investigated if the *miR-15a/16-1* cluster, tumor protein p53, and *miR-34b/c* cluster are connected in a molecular pathway that could explain the prognostic implications of 11q, 17p, and 13q deletions in CLL [32]. Several *TP53* binding sites were found upstream *miR-15a/16-1* on chromosome 13, *miR-34b/c* on chromosome 11, and *miR-34a* on chromosome 1. Thus, *TP53* could induce the expression of these microRNAs [32]. Besides, *miR-15a/16-1* target *TP53* while *miR-34* targets ZAP-70 mRNA expression [32]. In 13q deleted patients, the loss of *miR-15a/16-1* expression shifts the balance not only toward higher levels of anti-apoptotic Bcl2 [10,23] but also toward higher levels of pro-apoptotic p53. Consequently, the number of apoptotic cells decreases because of the lower level of Bcl2, but the intact p53 pathway keeps the tumor growth relatively low. This finding could explain how 13q deletions associate with indolent CLL. Moreover, increased p53 levels in patients with 13q deletions associate with transactivation of *miR-34b/c* leading to reduced levels of ZAP-70, positively correlating with survival [3]. CLL patients with 11q deletion, instead, show lower levels of *miR-34b/c* and higher levels of ZAP-70. In these patients, TP53 is not upregulated because *miR-15a/16-1* are not deleted and this condition is associated with lower control on apoptosis. TP53 transactivation of *miR-34b/c* is ineffective, since the locus is deleted, leading to a higher expression of ZAP-70 which correlates with poor prognosis [32] (Figure 1). 17p deletion and *TP53* mutation identifies the majority of chemotherapy-resistant patients. However, almost half of the refractory cases cannot be explained by a direct defect of p53. Zenz et al. studied *miR-34a* and *miR-34b/c* expression in refractory CLL with and without 17p deletion or *TP53* mutation [33]. While no expression of *miR-34b/c* was detected, downregulation of *miR-34a* was observed in 17p deleted and/or *TP53* mutated cases and in fludarabine-refractory cases even in the absence of 17p deletion/*TP53* mutation. Therefore, low expression of *miR-34a* in CLL is associated not only with p53 inactivation but also with chemotherapy-refractory disease, impaired DNA damage response, and apoptosis resistance, regardless of 17p deletion/*TP53* mutation [33].

#### *MicroRNA-181b* and *microRNA-29*

In both indolent and aggressive CLLs, *miR-29* is overexpressed when compared to normal B cells while *miR-181b* is downregulated when compared to normal B cells. Although, when comparing indolent and aggressive CLLs, both *mir-29* and *mir-181b* show higher expression levels in indolent cases [34-36]. Moreover, *miR-181b* expression is decreased during CLL progression when sequential samples from the same patients are compared [11]. In the same study, *miR-181b* is also reported to target *MCL1* in CLL, while in another study on human gastric and lung cancer cell lines, it was found able to target *BCL2* and involved in the development of multidrug resistance [37]. To clarify the role of *miR-29* in CLL, we designed a transgenic mouse model and mice overexpressing *miR-29* developed a disease similar to the indolent form of CLL. The role of downregulation of *miR-29* and *miR-181b* in aggressive CLLs appears to correlate with Tcl1 overexpression [35]. Activation of *TCL1* oncogene (T cell leukemia/lymphoma 1) is a central event in the initiation of aggressive CLL, and high Tcl1 expression correlates with aggressive phenotype [38]. We verified that coexpression of *TCL1* with *miR-29* and *miR-181* decreased its expression and found inverse correlation between *miR-29b*, *miR-181b*, and Tcl1 expression in CLL samples [35].

#### MicroRNA-17/92

*MiR-17/92* is a polycistronic microRNA cluster overexpressed in several lymphoid malignancies. In 2008, Xiao et al. [39] generated mice with higher expression of *miR-17/92* in lymphocytes. These mice developed lymphoproliferative disease and autoimmunity and died prematurely. Lymphocytes from these mice showed more proliferation and less apoptosis. *MiR-17/92* inhibited the expression of the tumor suppressor PTEN and the proapoptotic protein Bim. These data indicate that PTEN and Bim were direct targets of *miR-17/92* cluster molecules [39].

A recent study demonstrated that BCR signals can modulate expression of microRNAs. Evidence that BCR response in aggressive UM-IgH V_H_ CLL is accomplished through upregulation of *miR-17/92* has been described. In this study, the BCR-driven induction of *miR-17/92* in UM-IgH V_H_ CLLs was proposed as possible regulator of B-cell proliferation/survival by downregulating anti-proliferative and/or pro-apoptotic genes [40].

To determine whether *miR-17/92* overexpression induces lymphomagenesis, we generated a transgenic mouse overexpressing *miR-17/92* in B cells [41]. Eighty percent of *mir-17/92* transgenics developed a B-cell malignancy characterized by expansion of CD19+ B cells. Forty-four microRNAs and 680 genes were differentially expressed in these malignant B cells compared to controls. Eight upregulated miRs were correlated with 101 downregulated target mRNAs, and 11 downregulated miRs were correlated with 66 upregulated target mRNAs. *ABCC3*, a member of the ABC transporter family involved in anticancer agents transport, phosphoinositide 3-kinase (*PI3K*), growth arrest, and DNA damage 45 (*GADD45*), IL-4 were among downregulated mRNAs in *miR-17/92* mice [41].

#### MicroRNA-155

*MiR-155* and *BIC*, its host gene, have been reported to accumulate in human B-cell lymphomas, especially in diffuse large B-cell lymphomas, Hodgkin lymphomas, and certain types of Burkitt lymphomas [42]. To study its role in B-cell development and lymphomagenesis, we generated a transgenic mouse expressing *mmu-miR-155* in B cells [42]. We showed that Eμ-*mmu-miR-155* mice exhibit initially a preleukemic pre-B-cell proliferation evident in the spleen and bone marrow, followed by frank B-cell malignancy resembling the human diseases. These findings indicate that *miR-155* is able to induce polyclonal expansion, favoring the capture of secondary genetic changes for full transformation and suggests that *miR-155* is directly implicated in the initiation and/or progression of these diseases [42].

It has been reported that expression of *miR-155* can alter the signaling pathways triggered by BCR response by modulating the expression of *SHIP1* in CLL. *SHIP1* encodes for Src homology-2 domain containing inositol 5-phosphatase 1, a phosphatase that may suppress surface immunoglobulin and BCR signaling. *SHIP1* is a predicted target of *miR-155*, and transfection experiments confirmed the targeting by reducing its expression and enhancing cell sensitivity to BCR stimulation. Survival signals from auxiliary cells in the microenvironment might contribute to the upregulation of *miR-155.* Indeed, T cells or accessory cells of the lymphoid tissue can enhance expression of *miR-155* by stimulating the BCR response via CD154/CD40 or *BAFF/APRIL* interaction and *in vitro* CLL cells stimulated with CD154 or *BAFF/APRIL* show reduced expression of *SHIP1* and enhanced responsiveness to BCR ligation. Similar effects were also noted in normal B cells, indicating that *miR-155* might play a physiologic role in regulating the B-cell response to BCR ligation [13].

Another microenvironmental system that has recently been studied is represented by the role of microvescicles (MVs) in intercellular communication [6]. Recently, Ferrajoli et al. [17] hypothesized that some miRNAs might be involved in the transition from monoclonal B-cell lymphocytosis (MBL) to CLL and that these miRNAs may also predict response to therapy. They found *miR-155* increasingly overexpressed both in cells and in circulating microvesicles (MVs) from normal to MBL and CLL samples. Furthermore, *miR-155* expression was higher in non-responder than responder patients and the plasma level of *miR-155* was lower in patients who achieved complete remission than in patients who experienced other responses. These data validate the ability of *miR-155* to predict response to treatment and suggest that increasingly higher expression of *miR-155* from normal B cells to MBL to CLL and its overexpression in the plasma can be used as a predictor for poor response to treatment and disease progression.

## Conclusions

The ability of microRNAs to modulate gene expression is essential to provide fine control on several cell processes and its deficiency can be involved in CLL development/progression. Indeed, their deregulation can affect downstream pathways on cell cycle and proliferation. *MiR-15a/16-1* deletion is an initializing step in CLL, eliciting the control on Bcl2 expression. *MiR-34* family members are involved in a fine-regulated feedback circuitry with p53 and *miR-15a/16-1* in 13q deleted CLL, suggesting bidirectional interplay between microRNAs and genes. Moreover, restoring expression of *miR-15a/16-1* indirectly affects expression of *miR-34* family by modulating p53 expression and downregulation of *miR-29 and miR-181b* in aggressive CLL contributes to overexpression of Tcl1 [43]. Furthermore, study of *miR-17/92* and *miR-155* may provide useful insights into drug design, delivery, resistance mechanisms, and microenvironmental responses [17,41]. BCR stimulation can alter the expression of certain microRNAs, and BCR-regulated microRNAs might affect B-cell proliferation and apoptosis [14]. Further studies could aim to develop new therapies that disrupt the capacity of CLL cells to home to tissue microenvironments or that inhibit the signaling from accessory cells within the microenvironment. For example, agents that block the capacity of CLL cells to engage accessory cells might be expected to decrease the levels of *miR-155*, which increases cell sensitivity to BCR [13]. Lastly, deregulation of microRNAs can originate from chromosomal alteration, epigenetic modulation, allele selection, aberrant precursor processing, or interaction with other genes. Given these results, we can underline that microRNAs have a deep impact on CLL development/progression.
